# East London Hospital for Children, and Dispensary for Women

**Published:** 1893-10-21

**Authors:** Samuel Whitford

**Affiliations:** Secretary


### EAST LONDON HOSPITAL FOR CHILDREN, AND DISPENSARY FOR WOMEN.

Sir,—In your article in The Hospital for September 9th, in which you comment on the expensive management of the East London Hospital for Children, there is a mistake (owing to one made by our clerk in a return to you) as to facts which is of great importance, and I would beg you to correct it on the ground of its being misleading to the public and prejudicial to the interests of the hospital, situated as it is in one of the poorest parts of London, seldom visited by the wealthy, and supported with great difficulty, and managed as the board esteem with the greatest economy compatible with efficiency. The mistake I refer to is the statement that the number of out-patients admitted last year was 7,296—the correct figure is 19,577 (7,296 was the number of new out-patients admitted on letter, but we attend all who come even without). I would also beg to remind you that this hospital stands alone, I believe, among those to which you refer, in admitting infants without limit of age or number; one ward of over thirty beds is indeed constantly full of young infants, many of them but a few days old. A considerably larger staff of nurses is thus absolutely necessary than would otherwise be the case.—I am, Sir, yours faithfully,

Samuel Whitford, Secretary.

